# Adaptation of wild boar (*Sus scrofa*) activity in a human-dominated landscape

**DOI:** 10.1186/s12898-019-0271-7

**Published:** 2020-01-09

**Authors:** Franz Johann, Markus Handschuh, Peter Linderoth, Carsten F. Dormann, Janosch Arnold

**Affiliations:** 1 Department of Biometry and Environmental System Analysis, Albert-Ludwigs-University, Freiburg i. Br., Germany; 2 Wildlife Research Unit, Agricultural Centre Baden-Württemberg, Aulendorf, Germany; 3 Chair of Wildlife Ecology and Management, Albert-Ludwigs-University, Freiburg i. Br., Germany

**Keywords:** Circadian activity pattern, Nocturnal, Diurnal, Hunting, Light–dark circle, Behavioural flexibility, Disturbance, Wild boar

## Abstract

**Background:**

Wild boars (*Sus scrofa* L.) are globally widely distributed, and their populations have increased in Europe during recent decades. Encounters between humans and wild boars are rare because of the predominantly nocturnal lifestyle of the latter, and wild boar management by hunting is a challenging task. Animal activity patterns are important for understanding the behaviour of a species. However, knowledge of detailed temporal patterns and an understanding of the drivers of wild boar activity at a fine temporal scale are lacking. Of special relevance for human–wild boar interactions (e.g., encounters, conflicts, and management) is the question of whether nocturnal activity depends on anthropogenic factors and, particularly, how local hunting regimes may affect activity patterns. We used GPS telemetry and acceleration measurements to shed light on this part of wild boar behaviour, observing 34 animals in Central Europe. Animals were tracked along a gradient of hunting pressure from hunting-free areas to areas with low or high hunting pressure. Fitted generalised additive models allowed predicting the probability of active behaviour under differing disturbance regimes precisely to day of year and time of day.

**Results:**

The wild boars were predominantly nocturnal, with peak activity at approximately midnight. However, the data showed increased activity during daylight for wild boars that used no-hunting zones or reduced-hunting zones. Large areas with low disturbance levels promoted activity during daylight more than smaller areas with an intermediate disturbance regime. High air temperatures and locations within forests reduced the probability of active behaviour, whereas proximity to tracks used for forestry or agriculture was accompanied by a higher probability of activity.

**Conclusions:**

We conclude that wild boars flexibly adjust their activity to their local environmental conditions, considering disturbances at the scale of long-term home ranges as well as actual small-scale landscape quality. Entire wild boar home ranges should be covered in the delineation of reserves intending to stimulate activity during daylight.

## Background

Wild boar (*Sus scrofa* L.) is widely distributed, not least because of anthropogenic translocations of animals for supplying meat for human consumption [1, 2]. In Europe, wild boar has been endemic for millennia [3], and prehistoric cave art even documented the importance of the species for humans [4]. Wild boar is one of largest free-living terrestrial mammals in Central Europe, and the species is gaining particular attention from policy and wildlife managers for several reasons. The high reproductive rate [5], great adaptiveness to different environments [6] and widespread lack of predators of wild boar have favoured increases in its populations as well as expansion of its distribution ranges in Europe [7]. Climate change provides more abundant and more frequent oak and beech masts [8]—a coveted wild boar diet—and is therefore discussed as a relevant driver of growing wild boar populations [9–11].

The increased wild boar populations raise concerns in several respects. Wild boar functions as a vector of diseases that can affect livestock. Recently, African swine fever (ASF) spread into several areas of Europe [12]. This contagious viral disease affects wild boars as well as domestic pigs [13]. An outbreak into domestic pig farming would cause severe losses of animals, accompanied by economic losses for meat producers through collapsing markets. Furthermore, wild boars use agricultural areas as feeding grounds or resting sites, causing agricultural damage; yearly compensation payments in Europe amount to several million Euros [7, 14, 15]. Economic losses for grain growers or compensation expenses for wild boar destruction would probably be reduced at lower wild boar densities [16]. Similarly, a low wild boar density is seen as a means of reducing ASF risk [17]. Hunting of wild boar has a long tradition in Europe both for the procurement of meat and other resources and as an instrument for reducing human–wild boar conflicts. However, human activities such as hunting, recreation, agriculture and forestry affect the behaviour of many taxa, including ungulates [18–20]. It has been shown that anthropogenic disturbances not only cause changes in the spatial habitat use of wild boar and other ungulates [21–23]; they also modify the circadian patterns of activity towards more nocturnal behaviour in ungulates and other mammals [24–26]. In Europe, hunting is the major cause of wild boar mortality in unprotected populations [27, 28] and may be a crucial driver of wild boar activity patterns.

Because of the mostly nocturnal activity of wild boar [29, 30], sightings of wild boars are rare despite their increasing populations. However, wildlife sightings are desired by many people (see [31]) and may contribute to human well-being [32, 33]. If hunting reduces wild boar activity during daylight, a modification of wild boar management to facilitate positive wild boar experiences is an objective that should be discussed.

In addition to the likelihood of human–wild boar encounters, spatio-temporal activity patterns of wild boars have manifold desired or undesired environmental ramifications by determining the time and place of processes such as interactions with other species, consumer-resource interactions, translocations of nutrients, or the distribution of diaspores [34, 35]. A detailed knowledge of these spatio-temporal activity patterns may help assess and control their consequences.

Understanding wild boar activity patterns and evaluating human effects on wild boar activity are crucial for improving wild boar management. Several studies of wild boar activity have been conducted based on radio telemetry [6, 29, 36–40], acoustical detection [41] or use of camera traps [42, 43]. Because these methods are labour-intensive, the sampling frequency is low in comparison to the potential frequency of GPS collars with built-in accelerometers, and existing studies predominately compared activity levels at classified time spans such as night versus day or between seasons (but see [39–41]). However, activity patterns analysed at finer temporal scales based on frequent daily measurements may allow new insights into wild boar behaviour. Only recently Brivio et al. [30] analysed drivers of diurnal mean activity and nocturnal mean activity, based on GPS and accelerometer measurements and related environmental attributes.

Our goal was to reveal activity patterns and analyse their drivers at a finer temporal resolution, precise to day and time. We were particularly interested in the effect of hunting restrictions on the activity pattern. We expected wild boars to be more active at night [30, 41]. However, as previous studies found high daylight activity at a low human density [6, 43] and range shifts in response to hunting [22], we also expected noticeably increased activity during daylight in areas where hunting was restricted, compared to areas under a standard hunting regime. In hunting-free zones, we expected more activity during daylight than in zones where hunting was only reduced. In terms of the circannual pattern, we expected reduced activity during daylight throughout the main hunting period from November to February [44]. Furthermore, we expected a wild boar preference for undisturbed resting sites and consequently a lower activity level in forests and at greater distance from tracks.

## Results

The period of activity recording varied by individual from a minimum of 10 days to a maximum of 397 days with averages of 138.1 (standard deviation (SD) = 136.2) days for the Swabian Alps, 129.5 (103.9) days for Wurzach Marsh and 169.6 (167.0) days for Altdorf Forest. The number of wild boar individuals per month ranged from 9 to 21 with a mean of 15.6 (3.4).

**Fig. 1:**
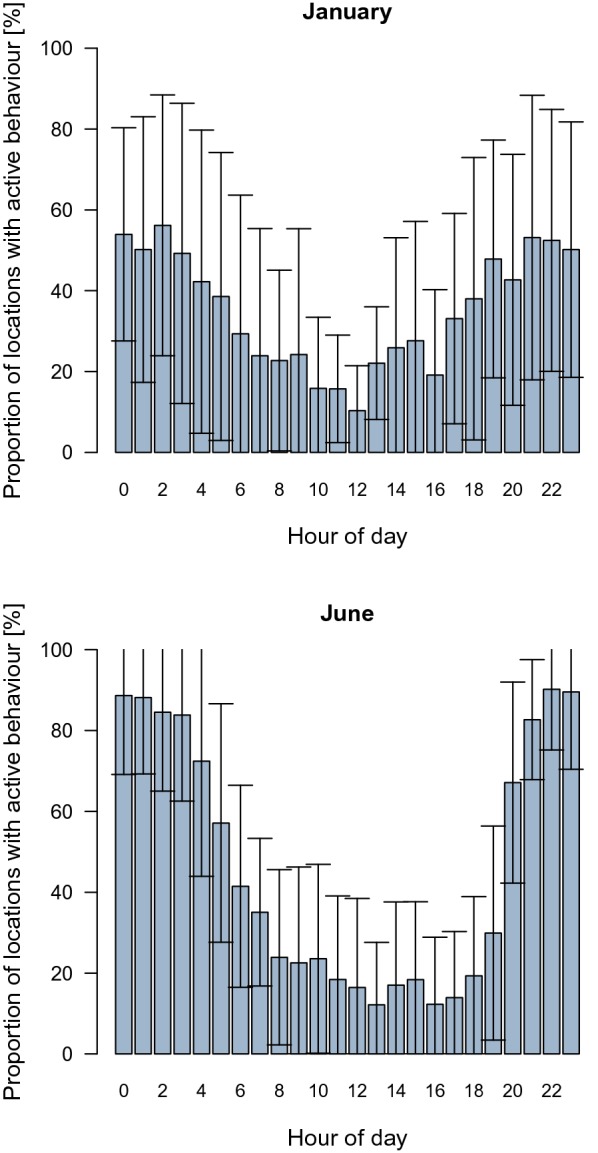
Percentage of locations with active behaviour depending on hour of the day. Means (error bars indicate 1 SD) of all wild boars from all three regions; *N*_*IDs January*_ = 13, *N*_*IDs June*_ = 19

The mean percentage of active behaviour over 24 h across all seasons and animals was 41.3% (SD = 5.4%, *N*_*IDs*_ = 34). The percentage changed over the course of the year and was highest in June at 46.2% (SD = 5.0%, *N*_*IDs*_ = 19), and lowest both in January and in February at 35.1% (SD = 8.4%, *N*_*IDs*_ = 13) and 35.1% (SD = 7.4%, *N*_*IDs*_ = 14), respectively. When separated by hour and month, the data revealed strong changes in the average proportion of locations with active behaviour over the course of the day. Peaks of activity occurred at approximately midnight. The differences between activity during night hours and activity during day hours were least distinct in January, primarily because of low activity at night; however, activity during daylight was also increased in comparison to that in June (Fig. 1).

### Time-of-day models

The Time-of-day (ToD) models confirmed a unimodal circadian activity pattern for all three regions. The fits for mid-January showed, in comparison to those for mid-April, mid-July and mid-October, markedly higher activity during daylight and lower activity levels at night in all regions.

**Fig. 2:**
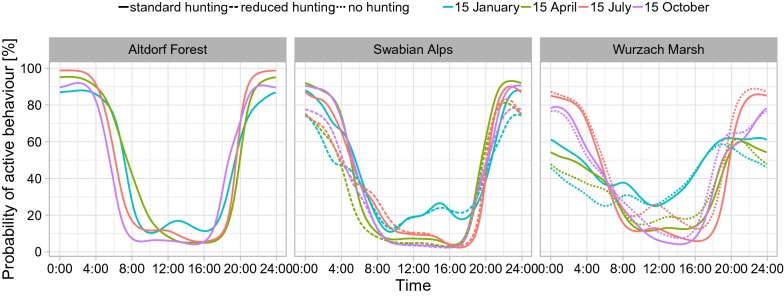
Probability of active behaviour depending on time of day, date and hunting pressure. Regions: Altdorf Forest (left), Swabian Alps (middle) and Wurzach Marsh (right); estimates from ToD models. Each line represents the mean across all collared animals in that region

In the Swabian Alps region, the activity level at night was lower in the reduced-hunting zone than in the standard-hunting zone, whereas little difference was found during the day (Fig. 2, middle).

In the Wurzach Marsh region, the probability of activity during night hours was lower in the hunting-free zone than in the standard-hunting zone in mid-January and mid-April, but in mid-July and mid-October it was approximately the same in both zones. When comparing the estimates of the probability of active behaviour during daylight between the different hunting zones of Wurzach Marsh, daylight activity was higher in the hunting-free zone than in the standard-hunting-zone in mid-April, mid-July, and mid-October. The difference was greatest in mid-July. In mid-December the daylight activity levels were mostly equal with increased activity in the standard hunting zone during the morning hours (Fig. 2, right).

### Phase-of-day models

**Fig. 3:**
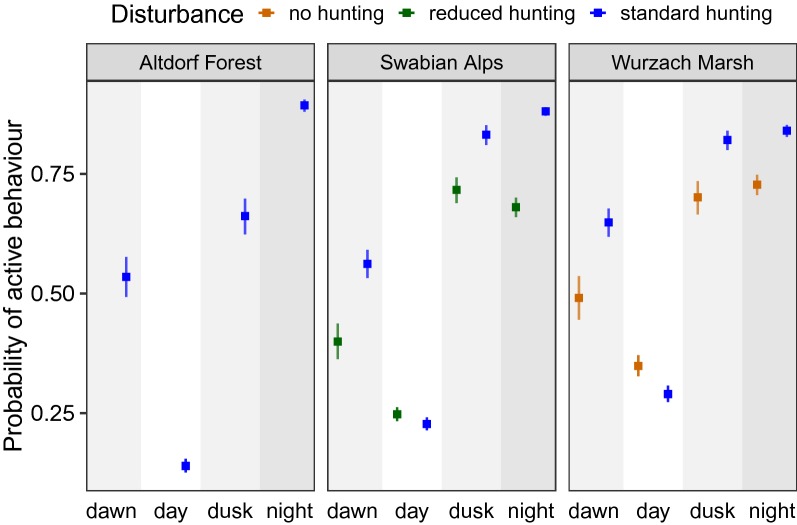
Estimated marginal means of the probability of active behaviour depending on region and disturbance; reduced phase-of-day models, error bars indicate 95% confidence intervals

The reduced phase-of-day (PoD) models showed the lowest activity at daylight, followed by dawn, dusk and night, for all regions. When comparing regions, the activity levels between night and daylight were most different in Altdorf Forest. In the Swabian Alps, activity during daylight was slightly higher in the reduced-hunting zone than in the standard-hunting zone. Similarly, in Wurzach Marsh, daylight activity was higher in the no-hunting zone than in the standard-hunting zone, whereby the difference between the two daylight activity levels was slightly greater in Wurzach Marsh than in the Swabian Alps (Fig. 3, for model coefficients see Additional file 1: Tables S1–S3). Wild boar identity had a significant effect (p < 0.001) in all three regions. The AIC of the reduced PoD models was higher than the AIC of the full PoD models (Altdorf Forest: 17,704 vs. 16,632, Swabian Alps: 46,937 vs. 44,432, Wurzach Marsh: 45,707 vs. 42,829).

In the full-phase-of-day models, the influence of the predictor terms on the probability of active behaviour differed between the three regions (Table 1). Phase of day, day of year and air temperature were always among the most relevant factors according to the *χ*^2^ values. The effect of hunting pressure ranked fourth and fifth in Wurzach Marsh and the Swabian Alps, respectively, in the regions where two levels existed. Distances to tracks and to forest edges as well as age class were sometimes important (Table 1). Wild boar identity had a significant effect (p < 0.001) in all three regions. An overview of all model coefficients is presented in Additional file 1: Tables S4–S6.

**Table 1:** Ranks 1 to 8 of the predictor terms of the full PoD models based on *χ*^2^

Rank based on *χ*^2^	Predictor term	*χ*^2^
Altdorf Forest		
1	Phase of day	2552.4
2	Day of year × PoD = night	314.8
3	Day of year × PoD = daylight	283.8
4	Day of year × PoD = dusk	282.1
5	Day of year × PoD = dawn	160.9
6	Air temperature	152.1
7	Elevation	88.5
8	Exposition	56.1
Swabian Alps		
1	Phase of day	1882
2	Air temperature	534.3
3	Day of year × PoD = daylight	268.7
4	Day of year × PoD = dusk	218.2
5	Hunting pressure = standard hunting × PoD	213.4
6	Distance to the next track	161.7
7	Wild boar identity	159.5
8	Exposition	156.3
Wurzach Marsh		
1	Air temperature	486.6
2	Land use type	337.0
3	Day of year × PoD = daylight	292.6
4	Day of year × hunting pressure = no hunting	277.5
5	Day of year × hunting pressure = standard hunting	217.0
6	Wild boar identity	196.5
7	Elevation	159.6
8	Distance to the next road	153.2

× indicates an interaction term. All terms were highly significant (p < 0.001)

**Fig. 4:**
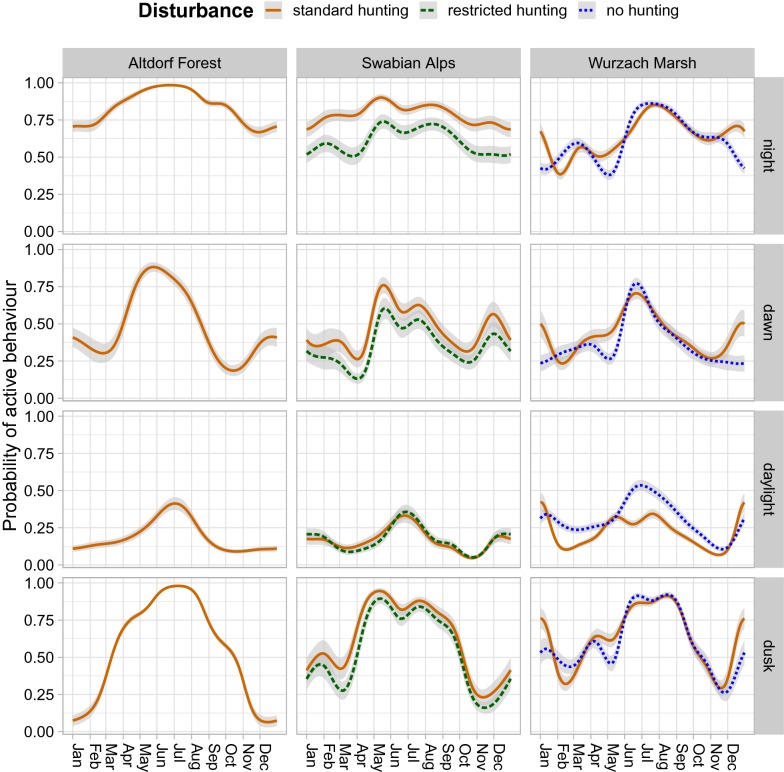
Probability of active behaviour depending on day of year, phase of day, region and hunting pressure. Night (top), dawn (second row), daylight (third row) and dusk (bottom); full PoD models; undepicted explanatory variables were set to the median or most common category. Shaded areas indicate one SD. Cyclic splines were fit for year (see Table 2 for more information on these models)

Over the course of the year, the probability of active behaviour was higher near the middle of the year. This variation was more pronounced at dawn and dusk and less distinct during daylight and at night. In the Swabian Alps region, the probability of active behaviour was lower in the reduced-hunting zone than in the standard-hunting zone, except during daylight when the reduced-hunting zone had slightly higher or equal activity levels from July to February. In contrast, in the Wurzach Marsh region, a clearly higher probability of activity during daylight was predicted for the no-hunting zone in comparison to the standard-hunting zone during most of the year, except for December and January. At the beginning of the main hunting season in November, daylight activity was low under all hunting regimes in the Swabian Alps and Wurzach Marsh; during December and January, it was higher again (Fig. 4, Table 2).

**Table 2:** Variables for predicting the probability of active behaviour of the full ToD models and full PoD models; separate models were fit for each of the three regions

Predictor	Information
Age class	Adult female, adult male, sub-adult female, sub-adult male, piglet
Air temperature	Hourly measurement in °C
Hunting pressure	Altdorf Forest: standard hunting Swabian Alps: standard hunting, restricted hunting Wurzach Marsh: standard hunting, no hunting
Time-of-day or phase-of-day	Second of day or dawn, daylight, dusk, night
ID	Wild boar identity
Day of year	1 to 365
Land use category	Forest, agriculture, bog, others
Distance to forest edge	Next forest edge, at locations in forest noted as negative values
Distance to road	Distance to the next road
Distance to track	Distance to the next forest track or field road
Exposition	Northeast, east, southeast, south, southwest, west, northwest, north
Slope	Slope in degrees
Elevation	Elevation above sea level
Moon-phase	Theoretical moon visibility, 0 to 100 percent
Weekend	True/false (Friday 5 pm to Sunday 12 pm)
Size of the hunting Free/restricted hunting area	Area in ha; for locations outside of protected areas the size of the closest protected area
No human access	True/false (only in models for the Swabian Alps)

**Fig. 5:**
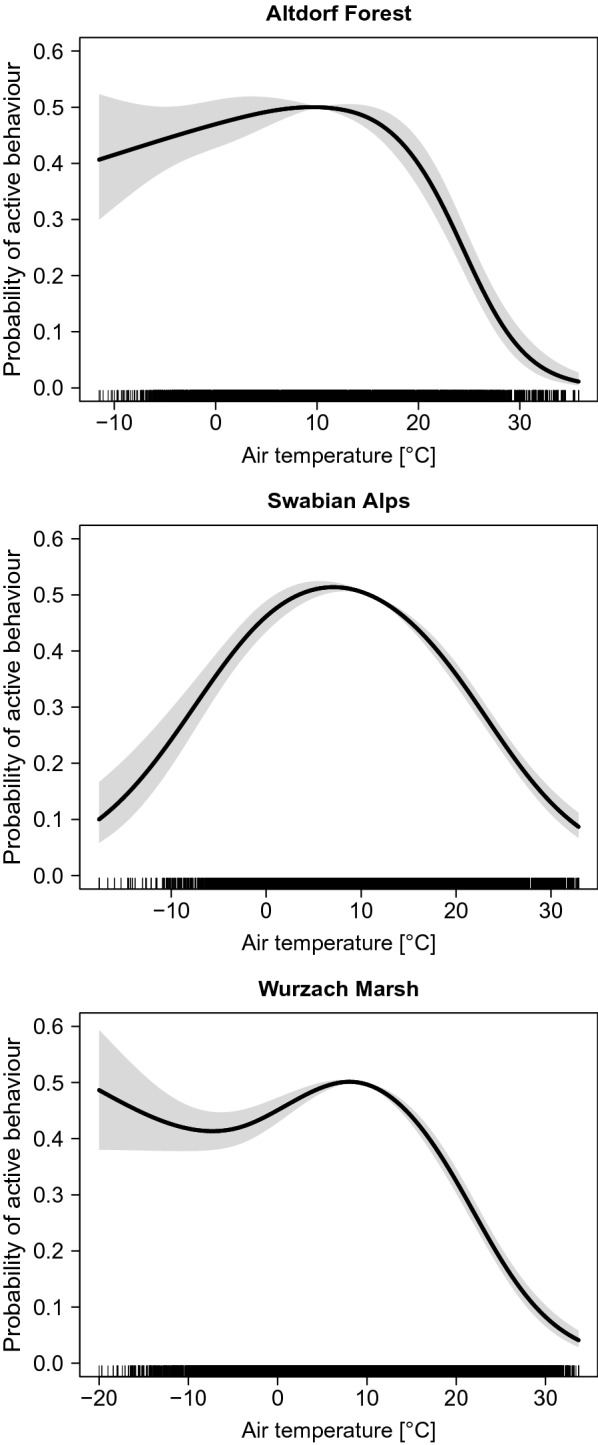
Effect of air temperature on the probability of active behaviour, full PoD models, undepicted explanatory variables were set to the median or most common category

Between temperatures of 0 °C and 17 °C, the probability of active behaviour was high in all of the regions. At higher temperatures, wild boars strongly reduced their activity. If the temperatures decreased below 5 °C, the probability of active behaviour decreased only slightly at in Altdorf Forest and Wurzach Marsh but strongly in the Swabian Alps (Fig. 5).

**Fig. 6:**
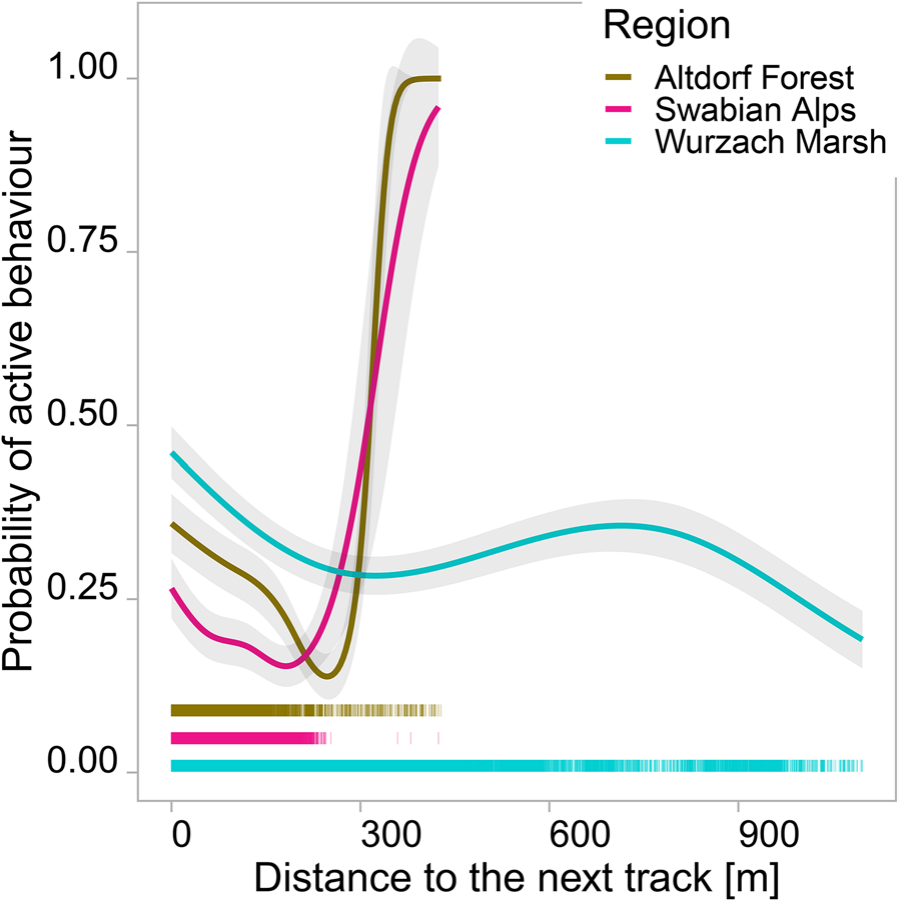
Estimated probability of active behaviour as a function of distance to track. Shades indicate one SD; rugs show observed distances; note that there are only few observations of great distances to tracks in Altdorf Forest and in the Swabian Alps region; full PoD models, undepicted explanatory variables were set to the median or most common category

If the animals were farther from tracks, the probability of active behaviour decreased (Fig. 6).

**Fig. 7:**
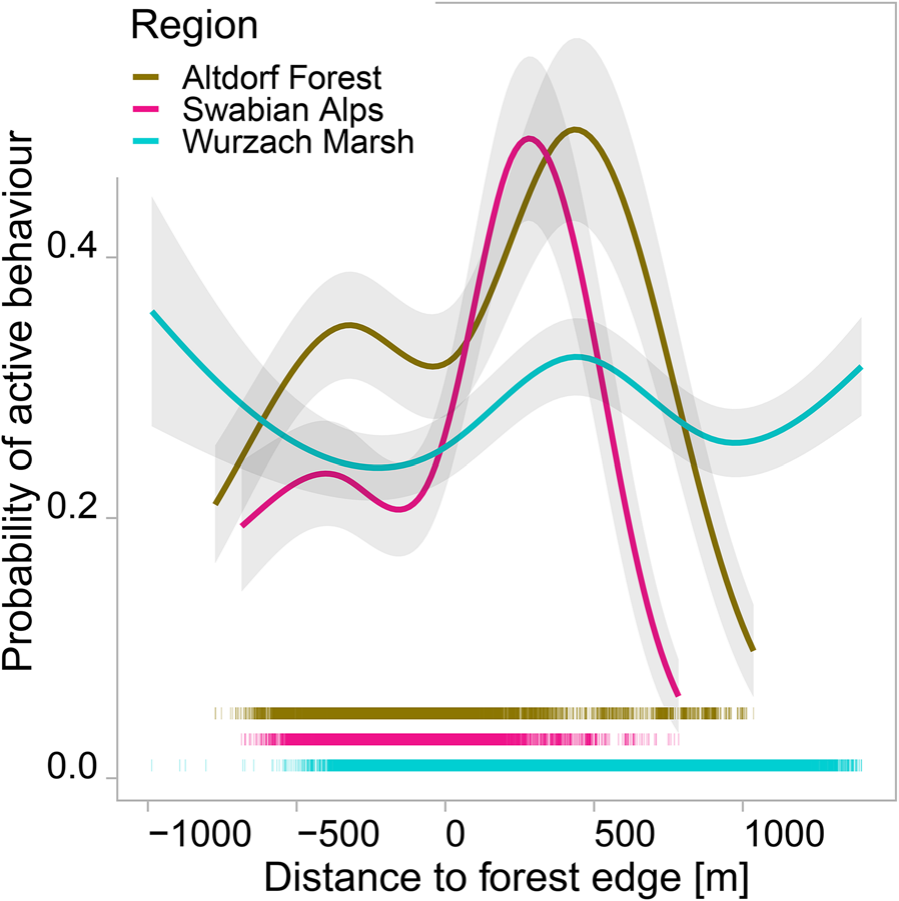
Estimated probability of active behaviour depending on distance to the next forest edge. Shades indicate one SD; negative distances indicate distances from inside the forest; rugs show observed distances, note that there are only few observations of great distance from forest edge in Altdorf Forest and in the Swabian Alps region; full PoD models

In all three regions, the estimated probability of active behaviour was lower in forests than for other land-use types (see Additional file 1: Tables S4–S6). The probability of active behaviour increased as wild boars left the forest; this effect was less pronounced in Wurzach Marsh than in the other regions. The locations of particularly low activity were inside the forest and near the forest edge (Fig. 7).

## Discussion

Our data revealed a strong day-night pattern, usually with one activity peak in the middle of the night. Pronounced variability in terms of activity was detected between individuals; in all models, the variable wild boar ID had a significant impact (p < 0.05). This result is consistent with population-level behaviour where some animals start being active earlier, others start later, and an inverse pattern occurs at the end of the main activity period at the end of the night. In the middle of the night, most individuals are active. Brivio et al. [30] described predominantly nocturnal and monophasic activity of wild boar in the Apennine Mountains, Italy, based on data collected by means of accelerometers on GPS collars. Boitani et al. [29] reported an activity level of 77.8% during the night and 30.1% during daylight, with a minimum at 3 p.m. for wild boars in Tuscany, Italy. In our study only Altdorf Forest and the standard hunting Zone of Swabian Alps had such high activity levels at night for large parts of the year (see Fig. 4).

According to our data and seen from a year-round perspective, wild boars spend on average more than half of the day resting. However, the activity levels during the four phases of a day vary in the course of the year with higher probability of active behaviour during the vegetation period.

During cold and long winter nights, the activity level is relatively low, whereas during short summer nights, the probability of activity is high. The nights being short probably forces wild boars to forage beyond dark, leading to a relatively high activity level during daylight. This seasonal pattern in our data matches with the findings of Keuling et al. [40] for wild boars in northeastern Germany. The estimates from the ToD models suggest that summer daylight activity mostly occurs in the early morning hours. Particularly in the Altdorf Forest region, we observed wild boars during summer in crop fields at daylight [45].

Our data showed a reduction in activity with temperatures above 15 °C. Wild boars lack functional sweat glands, and reducing activity, in addition to wallowing, is a behavioural adjustment for preventing hyperthermia [46]. Behavioural thermoregulation may also be a cause of the high level of activity during summer nights [30, 47–49]. In addition to thermoregulatory advantages, wild boars may prefer to be active during summer days in the colder early morning and late evening because encounters with humans are less likely. The different results between the regions in terms of response to decreasing temperatures may be caused by differing disturbances and food availability. The high explanatory power of the predictor air temperature in the full PoD models—the variable had the highest *χ*^2^ value or ranked second after the variable phase of day—shows a strong impact of air temperature on wild boar behaviour.

The lower probability of active behaviour in forests (Fig. 7) suggests that this vegetation type is perceived by wild boars as a safer environment and hence preferred for resting. Thurfjell et al. [50] found more wild boar damage in agricultural areas close to the forest edge than expected by chance. In the Wurzach Marsh region, forests have less of an effect on activity level, as wild boars can find cover in the reeds of the marsh. Dardaillon [51] indeed suggested marshes to be an optimal wild boar habitat.

High activity near tracks (Fig. 6) points to wild boar behaviour that minimises the risk of predation. According to our data, wild boars rest more in locations farther from tracks, probably because tracks are perceived as indicators of this risk [22].

Human outdoor activities affect animals in many ways [20]. In addition to changing the spatial distribution of animals [23, 52], anthropogenic disturbances have been suggested to shift activity patterns towards nocturnal activity. Evidence for this hypothesis was reported with regards to carnivorous and omnivorous predators [53–56] as well as for ungulates [57, 58]. Because of the high mortality rate caused by hunting in harvested populations [27, 28], appropriate behavioural responses to times of hunting increase the survival chances of wild boars.

Our study is the first to present a comparison of yearly and daily activity patterns of wild boars under different hunting regimes. The probability of active behaviour during daylight noticeably increased with the absence of hunting and other human activities in the Wurzach Marsh region. In contrast, zones of reduced hunting at the Swabian Alps only slightly promoted active behaviour during daylight. In addition to the lower disturbance level in Wurzach Marsh, this region also offers a larger area in which disturbances are restricted. The mean (± 1 SD) annual 100% minimum convex polygon home range (MCP100; [59]) of wild boars in the three regions was 4485 ha (± 4062 ha, N = 10; [60]). The largest reserve zones are 561 ha in Wurzach Marsh and 230 ha in the Swabian Alps. Thus, the animals face hunting within a large portion of their annual range. This is also true for monthly MCP100 ranges which average 1289 ha (± 1711 ha, N = 217; [60]). As wild boars commonly use reserve areas and standard-hunting areas, their activity patterns are shaped by risk assessments learned in both zones.

Our results substantiate the theory of reduced nocturnality of wild boar under reduced anthropogenic disturbance (see also [6, 40, 42, 47, 61]). Phenotypic plasticity allows species to adjust their temporal patterns to match local conditions and in turn increase their fitness [62, 63]. Wild boar populations increase [7] when the animals exhibit nocturnal activity, suggesting that the species is well adapted to this circadian rhythm. Nevertheless, physiological traits of *S. scrofa* and experimental research suggest that undisturbed wild boars would prefer to be active during daylight or exhibit cathemeral activity: the eyes of many mammals use a tapetum lucidum to reflect received light to the light-sensitive retinal cells and thereby increase visual capacities. With adaptation to dim-light environments, a species can benefit especially at dawn and dusk or under moonlight. Wild boar eyes are not equipped with this tissue [64]. Although this supports the theory of wild boar as a day-active species, some other strictly nocturnal mammals lack a tapetum lucidum [65]. Experimental research showed that pigs can better discriminate between social counterparts under higher light intensity (see [66]). The adaptation of wild boars to nocturnal activity is clearly not optimal. Rather, there is a strong indication that this circadian rhythm is a temporary behavioural adaptation, which wild boar performs excellently. Undisturbed *S. scrofa* in enclosures were day-active [67–69].

## Conclusions

Humans and wild boars have adopted different diel activity rhythms. This reduces the risk of road accidents, but also reduces the probability of positive experiences with wild boars. Sightings of wildlife are desired by many people [31], and encounters with animals can contribute to human well-being [32, 70, 71]. However, in terms of wild boars, humans have very ambivalent attitudes: from actively feeding them to fearing them [72]. Positive impressions—for instance, caused by observing the smartness or the social behaviour of wild boars—can hardly be experienced by average citizens. The nocturnal behaviour of wild boars may lead to a perception of wild boar as a species causing damage without offering benefits to society. Consequently, the reputation of wild boar is partly that of a notorious crop raider [73]. However, the more negative the reputation of a species is, the less likely society is to accept an economic loss caused by it, and calls for reducing the density of the species follow. To halt this mutual reinforcement of nocturnal activity and negative perception, wild boar management may want to consider not only regulating wild boar populations but also creating opportunities for positive wild boar experiences. In addition to the economic interests of land users and stock farmers and the related aspects of food safety, the cultural values and plain satisfaction of observing wild boars as well as the ecosystem functions of the species [74] must be considered. Because of the dense human population in the study regions and comprehensive human land use, the designation of large protected areas requires difficult policy processes and existing protected areas are small compared to reserves in less densely populated regions. The federal state of Baden-Württemberg had 305 inhabitants * km^−2^ in 2015 [75]. Appropriate wild boar population sizes, optimised hunting strategies, large enough no-hunting-zones (considering wild boar home ranges) and environmental education can be tools for the development trajectory.

Due to the rooting activities of wild boar, which alter soil processes and soil traits, the species is considered an ecosystem engineer. Additional effects on the environment include predation of vertebrates and invertebrates and effects on plant communities by consumption or seed dispersal [74]. Behavioural responses to anthropogenic disturbances may modify the effects of a species on the ecosystem [35]. For instance, crop consumption by wild boars during the night in agricultural areas and subsequent excretion near daytime resting sites in forests could influence decomposer communities and increase nutrient subsidies in forests. As little is known about the ecosystem functions of wild boar in its native range [74], research in this field may be warranted.

Moreover, we can ask if the temporal niche partitioning between humans and wild boars is “a last-resource mechanism of coexistence where other mechanisms fail” [76; p.172] and if it has evolutionary consequences. Optimisation of adaptation to darkness in wild boar may pose new challenges for humans.

## Methods

### Study areas

The animals were collared in three regions in southwestern Germany, approximately 30 to 80 kilometres north of Lake Constance: (1) Altdorf Forest, (2) the Swabian Alps and (3) Wurzach Marsh. The regions differed in the composition of land-uses and disturbance regimes (Table 3). In the Swabian Alps and Wurzach Marsh regions, wild boar had been collared in or close to zones with hunting restrictions. These hunting restrictions had been issued by nature protection authorities in order to allow more undisturbed wild boar behaviour and the resulting ecosystem processes. The regulations led to different levels of hunting pressure between areas of the three study regions and both between areas within the Swabian Alps study region and between areas within the Wurzach Marsh region. The disturbance levels are summarized in Table 3 and described in detail below.

**Table 3:** Hunting practices and human access in the study regions at a glance

Disturbance	Region				
	Altdorf Forest	Swabian Alps		Wurzach Marsh	
	Standard-hunting zone	Standard-hunting zone	Restricted-hunting zone	Standard-hunting zone	No-hunting zone
Solitary hunting	Yes	Yes	No	Yes	No
Battues	0–1 year^−1^	0–1 year^−1^	0–1 year^−1^	0–1 year^−1^	No
Human access	Yes	Yes	Partial	Yes	No

Standard practices of wild boar hunting in southwestern Germany include solitary hunting from raised hides with the aid of bait (mostly maize) and occasional battues with dogs and beaters in late autumn or early winter. Hunters are mostly foresters and licensed recreational hunters in state-owned forests and recreational hunters on private and community properties.

(1) In Altdorf Forest, the animals did not use restricted-hunting zones or hunting-free zones; hunting is practised according to standard regulations of the federal state of Baden-Württemberg. In the Altdorf Forest region, the mean hunting bag is low (0.9 wild boar * 100 ha^−1^ year^−1^) in comparison to that in other regions of the federal state. This suggests a relatively low wild boar density. (2) In the Swabian Alps region, the collared wild boars used the standard hunting zone and ten restricted-hunting zones, with areas ranging from 14 ha to 230 ha and a mean (standard deviation) of 78.2 (69.8) ha. In the restricted-hunting zones, single hunting of wild boar is forbidden, but battues are occasionally (maximum of once annually) carried out. Outside the restricted-hunting zones, the majority of the annual hunting bag is shot at baiting sites. The mean hunting bag in the Swabian Alps region is 2.0 wild boar * 100 ha^−1^ year^−1^. (3) In Wurzach Marsh, two neighbouring hunting-free zones extend over 561 ha and 144 ha, and are separated by only a minor road. The standard-hunting area (mean hunting bag 0.66 wild boar * 100 ha^−1^ year^−1^) surrounding the hunting free zones in Wurzach Marsh belongs to the regions with the lowest hunting bags of wild boar in Baden-Württemberg.

(1) In Altdorf Forest public access is not restricted, whereas (2) in the Swabian Alps study area, public access is partly prohibited within one of the zones of restricted hunting. (3) In Wurzach Marsh, public access is generally prohibited within the hunting-free zones. These regulations of public access exist predominantly for safety reasons because of boggy conditions in Wurzach Marsh and as a legacy of previous use for military training at the Swabian Alps. Nonetheless, some disturbances occur even if human access is restricted, e.g., due to research activities or in association with nature protection measures.

In the regions, westerly wind dominated climatic conditions with a mean annual temperature of 6–9 °C and mean annual precipitation of 800–1000 mm shape the vegetation at elevations ranging from approximately 500 m to 850 m above sea level [77, 78]. All the study regions are intensively used for agriculture and include forest patches. In a 5-km buffer around the collaring sites the proportion of forest is 31% in the Swabian Alps, 14% in Wurzach Marsh and 37% in Altdorf Forest. Cropland covers 17% in the Swabian Alps, 21% in Wurzach Marsh and 21% in Altdorf Forest. The share of grassland is 34% at Swabian Alps, 42% at Wurzach Marsh and 31% at Altdorf Forest. Only Wurzach Marsh includes considerable areas of bog (11%); these are mainly located in the no-hunting zone. The forests are dominated by European beech (*Fagus sylvatica* L.) and European spruce (*Picea abies* L.) with a larger proportion of broadleaf forest in the Swabian Alps. The forests in the restricted-hunting zones of the Swabian Alps and in the no-hunting zones in Wurzach Marsh are not managed for wood utilisation. Roe deer (*Capreolus capreolus* L.) is the sole ungulate besides wild boar; wolf (*Canis lupus* L.) and lynx (*Lynx lynx* L.) are absent in the study regions [79].

### Collection of data

We used GPS-locations and acceleration measurements of 34 wild boars during a 3-year study (December 2012 to December 2015) to analyse their activity patterns. The wild boars were trapped in wood-clad corral traps of approximately 30 m^2^ that were equipped with live cameras and remote-controlled gates. Maize was used as bait. To minimize health risks [80], we did not anaesthetize the animals, but single animals were separated into a net tunnel and held with their eyes covered by two or three persons. Caught animals were fitted with Vectronic Aerospace GPS Plus collars [81]. For welfare reasons, we collared only wild boars heavier than 30 kg. The collaring process took approximately five to ten minutes per animal, and the animals were released thereafter at the same place. The collar was removed after a wild boar had been shot during ordinary hunting practice or by using the automated or remote-controlled drop-off mechanism of the collar. All procedures were carried out in accordance with Section 8 subsection 1, of the animal welfare law of the federal state of Baden-Württemberg [82]. The required permission was obtained from the Regional Authority, Tübingen (permission # WFS1/12). The present report adheres to the ARRIVE guidelines for reporting animal research [83].

Five animals were collared in Altdorf Forest, 15 in the Swabian Alps and 14 in Wurzach Marsh. The animals were classified according to age and sex at collaring by experienced wildlife biologists. The classes were piglet, sub-adult for 1- to 2-year-old animals, and adult. Piglets advanced into the sub-adult class 90 days after collaring and sub-adults were reclassified as adults 180 days after collaring. Because the age classes were adjusted according to the time elapsed since collaring some individuals are represented in more than one age class. All age classes are represented: nine adult females, 15 sub-adult females, one adult male, 12 sub-adult males and 10 piglets.

We used a PostgreSQL database (https://www.postgresql.org/) and R software [84] for data processing and followed Urbano’s and Cagnacci’s [85] recommendations for data quality assessments. Locations recorded during the first 24 h after collaring were omitted. Only locations of the heaviest wild boar with the best tracking record were retained in the data in cases where two or more collared animals roamed together. Additionally, we reduced the transmitted locations to samples with approximately 1-h intervals to minimise bias due to autocorrelation or unequal location frequencies (Table 4).

**Table 4:** Number of hourly locations and observed animals

Region	Locations per zone			Number of wild boars
	Standard hunting	Restricted hunting	No hunting	
Swabian Alps	25,373	20,051		15
Wurzach Marsh	27,645		13,642	14
Altdorfer Forest	20,055	–		5
Sum	73,073			34

The landscape features were extracted for each location from a digital terrain model [86]. Hourly air temperature was assigned based on the measurements at the nearest meteorological station [87] and elevation-corrected by 1 °C per 100 m. We classified the phase of day (PoD) as night, dawn, daylight or dusk. Using the functions ‘sunriset’ and ‘crepuscule’ of the R package ‘maptools’ [88], we calculated times of sunrise, sunset, beginning of dawn and end of dusk, considering the time and coordinates of each location. Times from sunrise to sunset were classified as daylight. The classes dawn and dusk were assigned according to their nautical definition to wild boar locations within the timespans from a sun position of 12° below the horizon until sunrise and from sunset until a sun position of 12° below the horizon, respectively. The lengths of the twilight phases ranged from 67 to 101 min, depending on the location and day of year. ‘Phase of day’ and ‘air temperature’ were correlated with a Pearson coefficient of 0.34 to 0.35, depending on the region. The Pearson correlation between ‘time of day’ and ‘air temperature’ was 0.13.

The wild boars were equipped with Vectronic GPS plus collars, which included accelerometers. Acceleration is the change in directional velocity per unit time. The accelerometers measured sideward acceleration (x-acceleration) and forward–backward acceleration (y-acceleration) four times per second. These measurements in fractions of a second were automatically averaged by the devices over 300 s for some of the wild boars, but over 64 s for the others, due to device settings [81]. All averages were recorded with a timestamp. To link the activity data to the GPS locations and to harmonise the two different activity recording intervals, we assigned, as applicable, the 300-s average activity or the average activity over five periods of 64 s (5 * 64 s = 320 s) to the GPS location that was closest in time to the respective activity timestamp.

**Fig. 8:**
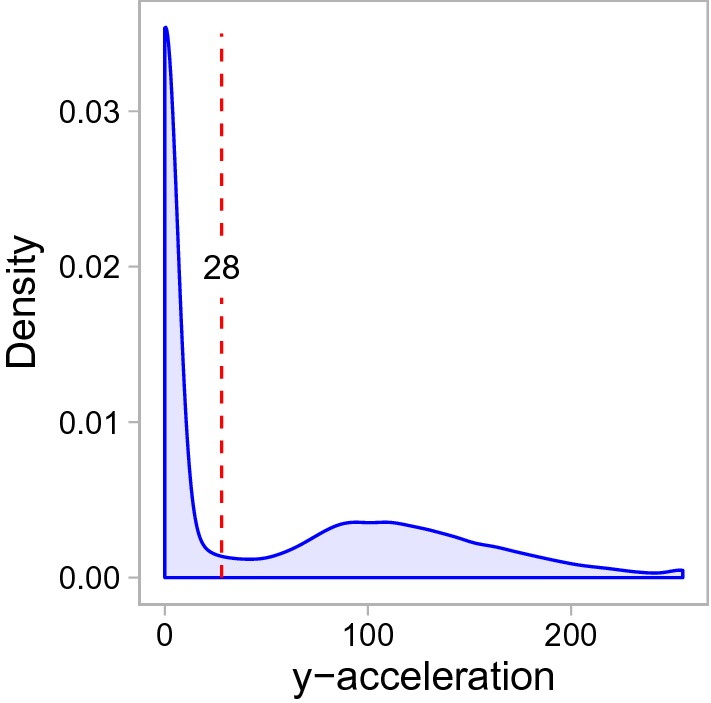
Density of the y-acceleration measurements. Density on a scale from 0 to 255 and applied threshold (dashed line) for classification of resting and active behaviours

As the x-acceleration and y-acceleration were correlated with a Pearson coefficient of 0.97 we used only the y-acceleration for further investigation. Activity was measured by the accelerometers in units of earth acceleration and transformed to an index with a scale from 0 (zero acceleration) to 255 (maximal acceleration) without a defined scale unit [written notification N. Gadow, Vectronic-Aerospace]. We classified the activity as resting up to a threshold of 28 and as active behaviour above this threshold, according to Thoma [89] (Fig. 8).

### Analysis of data

We calculated the percentage of active behaviour averaged over wild boar individuals (IDs) for 24-h periods and for 1-h intervals as first key-measurements. Furthermore, we fitted separate generalised additive models (GAMs) for each region by applying the R package ‘mgcv’ to analyse temporal activity patterns and the importance of predictors [90–92]. GAMs allow the analysis and depiction of non-linear, non-parametric relations between predictor and response variables by fitting complex regression curves. Because of the high behavioural plasticity of wild boar [6], we presumed complex, non-linear responses of activity levels along the gradients of external factors. Moreover, GAMs allowed cyclic smoothing terms for time of day and day of year as well as the inclusion of random effects to account for variability between wild boar individuals. GAMs are therefore an appropriate tool for the analysis of time-referenced data of several wild boar individuals. The response variable of the models was active behaviour or resting, coded as 0 (resting) and 1 (active). Consequently, the binomial models allowed predictions of patterns of active behaviour probability at the population level in the range of 0 (0% probability of active behaviour) to 1 (100% probability of active behaviour) along the gradients of the explanatory variables. We fitted complex multivariate time-of-day models (full ToD models) for each region, which included the time of day (ToD) of each location and analogous phase-of-day models (full PoD models), in which ToD was classified as phase of day (PoD) into the categories dawn, daylight, dusk and night. An overview of the predictors of the full PoD models and full ToD models is given in Table 2; details on the model structure are provided in Additional file 1. In addition, we fitted reduced phase-of-day models for each region to assess eventual bias due to confounding factors. The reduced models included the explanatory variables ‘day of year’, ‘phase of day by hunting level’ and additionally ‘wild boar ID’ as random effect.

Model performance was controlled using the function gam.check [92] and by comparing AIC values. To compare effect sizes, we calculated *χ*^2^-values by applying analysis of variance to the fitted model objects [93]. To compare activity levels, we estimated marginal means using the R package ‘emmeans’ [94] and we predicted activity levels along gradients of continuous explanatory variables using the packages ‘mgcv’ [92] and ‘visreg’ [95].

## Supplementary information

**Additional file 1.** Models, model formulas and model estimates of the PoD models for the regions Altdorf Forest, Swabian Alps and Wurzach Marsh.

## Data Availability

The datasets analysed during the current study are available from Janosch.Arnold@lazbw.bwl.de upon reasonable request.

## Abbreviations

AIC: Akaike Information Criterion
ASF: African swine fever
GAMs: generalised additive models
GPS: Global Positioning System
IDs: individuals
PoD: phase of day
SD: standard deviation
ToD: time of day
