# Supplementary material for: Adaptation of wild boar (Sus scrofa) activity in a human-dominated landscape
Source: BMC Ecol. 2020 Jan 9;20:4. doi: 10.1186/s12898-019-0271-7 (PMC6953143; doi:10.1186/s12898-019-0271-7)
Supplement: Supplementary file 1 — Additional file 1. Models, model formulas and model estimates of the PoD models for the regions Altdorf Forest, Swabian Alps and Wurzach Marsh. [file 12898_2019_271_MOESM1_ESM.pdf]

Models

Reduced phase-of-day-models

Formula:

activity_300s_320s_y_01 ~ nhz_phase + s(day_of _year, bs = "cc", k = 12) + s(animal_ID, bs = "re")

Table S1: model coefficients reduced phase-of-the-day-model Altdorf Forest region

**Parametric coefficients**					
**Predictor**	**Estimate**	**Std. Error**	**z value**	**P**	**Code**
intercept	-0.406	0.145	-2.808	0.005	**
standard hunting × daylight	-1.958	0.075	-26.063	< 0.001	***
standard hunting × dusk	0.532	0.094	5.657	< 0.001	***
standard hunting × night	1.987	0.076	26.270	< 0.001	***
**Approximate significance of continuous predictors and random effect**					
**Predictor**		**edf**	**Ref.df**	**P**	**Code**
s(day of year)		6.780	10	< 0.001	***
s(animal ID)		3.728	4	0.012	*

Code: *** < 0.001, ** < 0.01, *< 0.05

Table S2: model coefficients reduced phase-of-day-model Swabian Alps region

**Parametric coefficients**					
**Predictor**	**Estimate**	**Std. Error**	**z value**	**P**	**Code**
intercept	-0.736	0.108	-6.846	< 0.001	***
standard hunting × dawn	0.657	0.090	7.336	< 0.001	***
reduced hunting × daylight	-0.704	0.075	-9.383	< 0.001	***

standard hunting × daylight	-0.816	0.077	-10.630	< 0.001	***
reduced hunting × dusk	1.336	0.092	14.512	< 0.001	***
standard hunting × dusk	2.008	0.100	20.139	< 0.001	***
reduced hunting × night	1.163	0.077	15.118	< 0.001	***
standard hunting × night	2.408	0.076	31.538	< 0.001	***
**Approximate significance of continuous predictors and random effect**					
**Predictor**		**edf**	**Ref.df**	**P**	**Code**
s(day of year)		9.593	10	< 0.001	***
s(animal ID)		13.107	14	< 0.001	***

Code: *** < 0.001, ** < 0.01, *< 0.05

Table S3: model coefficients reduced phase-of-day-model Wurzach Marsh region

**Parametric coefficients**					
**Predictor**	**Estimate**	**Std. Error**	**z value**	**P**	**Code**
intercept	-0.727	0.100	-7.304	< 0.001	***
standard hunting × dawn	0.650	0.100	6.487	< 0.001	***
no hunting × daylight	-0.589	0.088	-6.702	< 0.001	***
standard hunting × daylight	-0.859	0.087	-9.827	< 0.001	***
no hunting × dusk	0.890	0.111	7.998	< 0.001	***
standard hunting × dusk	1.559	0.103	15.207	< 0.001	***
no hunting × night	1.019	0.089	11.443	< 0.001	***
standard hunting × night	1.698	0.087	19.520	< 0.001	***
**Approximate significance of continuous predictors and random effect**					
**Predictor**		**edf**	**Ref.df**	**P**	**Code**

s(day of the year)	9.728	10	< 0.001	***
s(animal ID)	11.418	13	< 0.001	***

Code: *** < 0.001, ** < 0.01, *< 0.05

Full phase-of-day-models

In the full PoD models, the categorical predictor terms are age class, hunting pressure, land-use type, weekend, human access and exposition; additionally, the interactions hunting pressure × phase of day and hunting pressure × no human access. Included simple continuous predictors are air temperature, slope, elevation, distance to forest edge, distance to roads, distance to tracks and moon-phase. Included interactions of continuous predictors are: (1) protected area size by hunting pressure, (2) protected area size by phase of day, (3) day of the year by hunting pressure, (4) day of year by phase of day, (5) and day of year by land use type. For all terms which comprise the predictor day of year we fitted cyclic smooth terms using bs = ‘cc’ and k = 12 to allow for a detailed fitting along the course of the year. Other continuous predictors were included allowing smooth shrinkage using bs = ‘cs’ and k = 5 as initial smooth setting. Additionally, we accounted for the variability between individuals by including wild boar identity as a random effect in the models. The time-of-day-models are structured analogously, but the predictor PoD is replaced by predictor ToD with the settings bs = ‘cc’ and k = 18.

Full phase-of-day-model Altdorf Forest region

Formula:

activity_300s_320s_y_01 ~ age_sex_actual_2 + naut_phase + s(day_of _year, by = naut_phase, bs = "cc", k = 12) + land_use_type4 + s(day_of_year, by = land_use_type4, bs = "cc", k = 12) + s(dist_to_forest_edge, bs = "cs", k = 5) + s(dist_track, bs = "cs", k = 5) + s(dist_road, bs = "cs", k = 5) + s(moon_percent, bs = "cs", k = 5) + s(slope, bs = "cs", k = 5) + s(elevation, bs = "cs", k = 5) + s(hourly_temp, bs = "cs", k = 5) + exposition + weekend + s(animal_ID, bs = "re")

Table S4: model coefficients full phase-of-day-model Altdorf Forest region

Parametric coefficients							Rank based on χ^2
**Predictor**	**Estimate**	**Std. Error**	**z value**	**P**	**Code**	χ^2	
intercept	-0.165	0.207	-0.798	0.425			
age_sex_actual_2_sub_adult_m	0.458	0.119	3.838	< 0.001	***	14.82	15
age_sex_actual_2_piglet	0.191	0.112	1.699	0.089	ns		
naut_phasenaut_day	-1.458	0.087	-16.782	< 0.001	***	2552.40	**1**
naut_phasenaut_dusk	0.5997	0.129	4.660	< 0.001	***		
naut_phasenaut_night	2.130	0.095	22.444	< 0.001	***		
land_use_type4_bog	-0.308	0.470	-0.656	0.512	ns	37.13	11
land_use_type4_bog	-0.273	0.453	-0.602	0.547	ns		
land_use_type4_forest	-0.6283	0.1033	-6.081	< 0.001	***		
expositionNO	-0.2003	0.0973	-2.054	0.040	*	56.14	**8**
expositionO	-0.2333	0.093	-2.511	0.012	*		
expositionSO	0.116	0.091	1.284	0.199	ns		
expositionS	0.252	0.095	2.653	0.008	**		
expositionSW	0.090	0.098	0.918	0.359	ns		
expositionW	-0.039	0.098	-0.399	0.690	ns		
expositionNW	-0.079	0.112	-0.707	0.479	ns		
weekend_t	-0.0003	0.042	-0.008	0.994	ns	0.00	20.5
**Approximate significance of continuous predictors and random effect**							
**Predictor**		**edf**	**Ref.df**	**P**	**Code**	χ^2	
s(day_of_year):naut_phasenaut_dawn		6.614	10	< 0.001	***	160.933	**5**
s(day_of_year):naut_phasenaut_day		6.310	10	< 0.001	***	283.757	**3**

s(day_of_year):naut_phasenaut_dusk	7.729	10	< 0.001	***	282.125	**4**
s(day_of_year):naut_phasenaut_night	8.203	10	< 0.001	***	314.820	**2**
s(day_of_year):land_use_type4_agriculture	9.390	10	< 0.001	***	40.289	10
s(day_of_year):land_use_type4_bog	0.003	4	0.567	ns	0.001	19
s(day_of_year):land_use_type4_others	4.120	10	0.246	ns	5.404	16
s(day_of_year):land_use_type4_forest	0.004	10	< 0.001	***	0.005	18
s(dist_to_forest_edge)	3.941	4	< 0.001	***	31.554	13
s(dist_track)	3.906	4	< 0.001	***	53.226	9
s(dist_road)	0.0006	4	0.427	ns	0.000	20.5
s(moon_percent)	2.736	4	0.113	ns	5.311	17
s(slope)	3.740	4	< 0.001	***	33.675	12
s(elevation)	3.983	4	< 0.001	***	88.521	**7**
s(hourly_temp)	3.894	4	< 0.001	***	152.112	**6**
s(animal_ID)	3.625	4	< 0.001	***	30.887	14

Variables may occur in more than one predicting term; code: *** < 0.001, ** < 0.01, *< 0.05; the first eight ranks based on χ^2 are printed in bold

Full phase-of-day-model Swabian Alps region

Formula:

activity_300s_320s_y_01 ~ nhz + age_sex_actual_2 + naut_phase * nhz + s(size_nhz, by = nhz, bs = "cs", k = 5) + s(size_nhz, by = naut_phase, bs = "cs", k = 5) + s(day_of_year, by = nhz, bs = "cc", k = 12) + s(day_of_year, by = naut_phase, bs = "cc", k = 12) + land_use_type4 + s(day_of_year, by = land_use_type4, bs = "cc", k = 12) + s(dist_to_forest_edge, bs = "cs", k = 5) + s(dist_track, bs = "cs", k = 5) + s(dist_road, bs = "cs", k = 5) + s(moon_percent, bs = "cs", k = 5) + s(slope, bs = "cs", k = 5) + s(elevation, bs = "cs", k = 5) + s(hourly_temp, bs = "cs", k = 5) + exposition + weekend + nhz * no_human_access + no_human_access + s(animal_ID, bs = "re")

Table S5: model coefficients full phase-of-day-model Swabian Alps region

**Parametric coefficients**							**Rank based on χ^2**
**Predictor**	**Estimate**	**Std. Error**	**z value**	**P**	**code**	χ^2	
intercept	-0.660	0.177	-3.740	< 0.001	***		
nhz_standard_hunting	0.482	0.109	4.437	< 0.001	***	19.687	17
age_sex_actual_2_adult_m	0.112	0.508	0.221	0.825	ns	43.912	13
age_sex_actual_2_sub_adult_f	0.195	0.040	4.928	< 0.001	***		
age_sex_actual_2_sub_adult_m	0.407	0.192	2.120	0.034	*		
age_sex_actual_2_piglet	0.884	0.191	4.638	< 0.001	***		
naut_phasenaut_day	-0.550	0.088	-6.260	< 0.001	***	1882.244	**1**
naut_phasenaut_dusk	1.560	0.109	14.277	< 0.001	***		
naut_phasenaut_night	1.256	0.090	13.891	< 0.001	***		
land_use_type4_bog	29.279	349.135	0.084	0.933	ns	17.443	20
land_use_type4_others	0.178	0.168	1.059	0.290	ns		
land_use_type4_forest	-0.213	0.058	-3.687	< 0.001	***		
expositionNO	-0.203	0.050	-4.073	< 0.001	***	156.279	**8**
expositionO	-0.226	0.050	-4.592	< 0.001	***		
expositionSO	0.225	0.049	-4.620	< 0.001	***		
expositionS	-0.323	0.056	- 5.778	< 0.001	***		
expositionSW	-0.515	0.053	-9.716	< 0.001	***		
expositionW	-0.502	0.049	-10.311	< 0.001	***		
expositionNW	-0.164	0.051	-3.197	0.001	**		
weekendt	0.108	0.025	4.315	< 0.001	***	18.619	18
no_human_accesst	0.155	0.089	1.731	0.083	ns	2.996	26

nhz_standard_hunting:naut_phasenaut_day	- 0.535	0.110	-4.881	< 0.001	***	213.422	**5**
nhz_standard_hunting:naut_phasenaut_dusk	- 0.078	0.150	-0.520	0.603	ns		
nhz_standard_hunting:naut_phasenaut_night	0.394	0.112	3.521	< 0.001	***		
nhz_standard_hunting:no_human_accesst	-0.133	0.112	-1.189	0.234	ns	1.414	27
**Approximate significance of continuous predictors and random effect**							
**Predictor**		**edf**	**Ref.df**	**P**	**code**	χ^2	
s(size_nhz):nhz_reduced_hunting		0.0264	4	0.119	ns	0.007	31
s(size_nhz):nhz_standard_hunting		2.882	4	0.004	**	12.672	23
s(size_nhz):naut_phasenaut_dawn		3.159	4	< 0.001	***	17.552	19
s(size_nhz):naut_phasenaut_day		2.354	4	< 0.001	***	74.662	11
s(size_nhz):naut_phasenaut_dusk		2.739	4	< 0.001	***	16.646	21
s(size_nhz):naut_phasenaut_night		1.841	4	0.003	**	9.350	24
s(day_of_year): nhz_reduced_hunting		0.0744	10	0.001	**	0.049	29
s(day_of_year): nhz_standard_hunting		6.899	10	< 0.001	***	24.377	16
s(day_of_year):naut_phasenaut_dawn		9.208	10	< 0.001	***	123.591	9
s(day_of_year):naut_phasenaut_day		9.480	10	< 0.001	***	268.700	**3**
s(day_of_year):naut_phasenaut_dusk		8.968	10	< 2e-16	***	218.177	**4**
s(day_of_year):naut_phasenaut_night		8.826	10	< 0.001	***	51.118	12
s(day_of_year):land_use_type4_agriculture		0.003	10	0.415	ns	0.002	32
s(day_of_year):land_use_type4_bog		0.623	6	0.399	ns	0.446	28
s(day_of_year):land_use_type4_others		0.002	10	0.618	ns	0.001	33
s(day_of_year):land_use_type4_forest		6.102	10	< 0.001	***	38.898	14
s(dist_to_forest_edge)		3.959	4	< 2e-16	***	115.523	10
s(dist_track)		3.860	4	< 2e-16	***	161.719	**6**
s(dist_road)		1.377	4	0.043	*	5.146	25

s(moon_percent)	1.224	4	< 0.001	***	15.510	22
s(slope)	3.905	4	< 0.001	***	26.104	15
s(elevation)	0.0132	4	0.304	ns	0.014	30
s(hourly_temp)	3.865	4	< 0.001	***	534.307	**2**
s(animal_ID)	12.128	13	< 0.001	***	159.457	**7**

Variables may occur in more than one predicting term; code: *** < 0.001, ** < 0.01, *< 0.05; the first eight ranks based on χ^2 are printed in bold

Full phase-of-day-model Wurzach Marsh region

Formula:

activity_300s_320s_y_01 ~ nhz + age_sex_actual_2 + naut_phase * nhz + size_nhz * nhz + size_nhz * naut_phase + s(day_of_year, by = nhz, bs = "cc", k = 12) + s(day_of_year, by = naut_phase, bs = "cc", k = 12) + land_use_type4 + s(day_of_year, by = land_use_type4, bs = "cc", k = 12) + s(dist_to_forest_edge, bs = "cs", k = 5) + s(dist_track, bs = "cs", k = 5) + s(dist_road, bs = "cs", k = 5) + s(moon_percent, bs = "cs", k = 5) + s(slope, bs = "cs", k = 5) + s(elevation, bs = "cs", k = 5) + s(hourly_temp, bs = "cs", k = 5) + exposition + weekend + s(animal_ID, bs = "re")

Table S6: model coefficients full phase-of-day-model Wurzach Marsh region

**Parametric coefficients**							**Rank based on χ^2**
**Predictor**	**Estimate**	**Std. Error**	**z value**	**P**	**Code**	χ^2	
Intercept	-0.847	0.431	-1.968	0.049	*		
nhz_standard_hunting	0.342	0.280	1.220	0.222	ns	1.489	25
age_sex_actual_2_sub_adult_f	0.112	0.064	1.759	0.079	ns	24.622	19
age_sex_actual_2_sub_adult_m	0.026	0.101	0.258	0.797	ns		
age_sex_actual_2_piglet	0.2898	0.010	2.892	0.004	**		

naut_phasenaut_day	-0.246	0.367	-0.670	0.503	ns	99.194	12
naut_phasenaut_dusk	1.998	0.469	4.261	< 0.001	***		
naut_phasenaut_night	1.376	0.358	3.840	< 0.001	***		
size_nhz	0.001	0.0007	1.972	0.049	*	3.887	24
land_use_type4_bog	-0.709	0.064	-11.137	< 0.001	***	337.031	**2**
land_use_type4_others	-0.171	0.097	-1.768	0.077107	ns		
land_use_type4_forest	-0.802	0.044	-18.122	< 0.001	***		
expositionNO	0.193	0.052	3.740	< 0.001	***	47.652	18
expositionO	0.020	0.046	0.441	0.659	ns		
expositionSO	0.022	0.049	0.453	0.651	ns		
expositionS	0.167	0.053	3.149	0.002	**		
expositionSW	-0.036	0.054	-0.657	0.511	ns		
expositionW	0.164	0.060	2.713	0.007	**		
expositionNW	-0.054	0.050	-1.092	0.275	ns		
weekendt	0.0595	0.025	2.354	0.019	*	5.543	22
nhz_standard_hunting:naut_phasenaut_day	-0.722	0.119	-6.065	< 0.001	***	97.848	13
nhz_standard_hunting:naut_phasenaut_dusk	-0.156	0.158	-0.988	0.323	ns		
nhz_standard_hunting:naut_phasenaut_night	-0.158	0.121	-1.305	0.192	ns		
nhz_standard_hunting:size_nhz	-0.0004	0.001	-0.844	0.399	ns	0.712	26
naut_phasenaut_day:size_nhz	-0.0003	0.001	-0.413	0.679	ns	4.997	23
naut_phasenaut_dusk:size_nhz	-0.002	0.001	-1.861	0.063	ns		
naut_phasenaut_night:size_nhz	-0.0006	0.001	-0.896	0.370	ns		
**Approximate significance of continuous predictors and random effect**							
**Predictor**		**edf**	**Ref.df**	**P**	**Code**	χ^2	

s(day_of_year):nhz_no_hunting	9.022	10	< 0.001	***	277.528	**4**
s(day_of_year): nhz_standard_hunting	9.686	10	< 0.001	***	217.009	**5**
s(day_of_year):naut_phasenaut_dawn	8.244	10	< 0.001	***	83.290	14
s(day_of_year):naut_phasenaut_day	9.844	10	< 0.001	***	292.591	**3**
s(day_of_year):naut_phasenaut_dusk	9.599	10	< 0.001	***	108.794	11
s(day_of_year):naut_phasenaut_night	0.011	10	< 0.001	***	0.011	28
s(day_of_year):land_use_type4_agriculture	6.160	10	< 0.001	***	143.235	9
s(day_of_year):land_use_type4_bog	4.734	10	0.001	**	14.269	21
s(day_of_year):land_use_type4_others	7.591	10	< 0.001	***	51.355	17
s(day_of_year):land_use_type4_forest	0.066	10	< 0.001	***	0.078	27
s(dist_to_forest_edge)	3.928	4	< 0.001	***	58.485	15
s(dist_track)	3.770	4	< 0.001	***	141.194	10
s(dist_road)	3.946	4	< 0.001	***	153.217	**8**
s(moon_percent)	3.762	4	< 0.001	***	18.337	20
s(slope)	3.855	4	< 0.001	***	52.330	16
s(elevation)	3.843	4	< 0.001	***	159.579	**7**
s(hourly_temp)	3.883	4	< 0.001	***	486.563	**1**
s(animal_ID)	9.972	13	< 0.001	***	196.478	**6**

Variables may occur in more than one predicting term; code: *** < 0.001, ** < 0.01, *< 0.05; the first eight ranks based on χ^2 are printed in bold
